# Sometimes Sperm Whales (*Physeter macrocephalus*) Cannot Find Their Way Back to the High Seas: A Multidisciplinary Study on a Mass Stranding

**DOI:** 10.1371/journal.pone.0019417

**Published:** 2011-05-18

**Authors:** Sandro Mazzariol, Giovanni Di Guardo, Antonio Petrella, Letizia Marsili, Cristina M. Fossi, Claudio Leonzio, Nicola Zizzo, Salvatrice Vizzini, Stefania Gaspari, Gianni Pavan, Michela Podestà, Fulvio Garibaldi, Margherita Ferrante, Chiara Copat, Donato Traversa, Federica Marcer, Sabina Airoldi, Alexandros Frantzis, Yara De Bernaldo Quirós, Bruno Cozzi, Antonio Fernández

**Affiliations:** 1 Department of Public Health, Comparative Pathology and Veterinary Hygiene, University of Padua, Legnaro, Italy; 2 Department of Comparative Biomedical Sciences, University of Teramo, Teramo, Italy; 3 Istituto Zooprofilattico Sperimentale della Puglia e della Basilicata, Foggia, Italy; 4 Department of Environmental Science, University of Siena, Siena, Italy; 5 Department of Veterinary Public Health, University of Bari, Valenzano, Italy; 6 Department of Earth and Marine Science, University of Palermo, Palermo, Italy; 7 Department of Evolutionary Biology, University of Firenze, Firenze, Italy; 8 Department of Animal Biology, Interdisciplinary Center for Bioacoustics, University of Pavia, Pavia, Italy; 9 Museum of Natural History of Milan, Milano, Italy; 10 Department for the Study Territory and its Resources, University of Genova, Genova, Italy; 11 Department of Anatomy, Diagnostic Pathology, Forensic Medicine, Hygiene and Public Health, University of Catania, Catania, Italy; 12 Department of Experimental Veterinary Science, University of Padua, Legnaro Italy; 13 Tethys Research Institute, Milano, Italy; 14 Pelagos Cetacean Research Institute, Vouliagmeni, Greece; 15 Department of Morphology, University of Las Palmas de Gran Canaria, Las Palmas, Spain; Biodiversity Insitute of Ontario - University of Guelph, Canada

## Abstract

**Background:**

Mass strandings of sperm whales (*Physeter macrocephalus*) remain peculiar and rather unexplained events, which rarely occur in the Mediterranean Sea. Solar cycles and related changes in the geomagnetic field, variations in water temperature and weather conditions, coast geographical features and human activities have been proposed as possible causes. In December 2009, a pod of seven male sperm whales stranded along the Adriatic coast of Southern Italy. This is the sixth instance from 1555 in this basin.

**Methodology/Principal Findings:**

Complete necropsies were performed on three whales whose bodies were in good condition, carrying out on sampled tissues histopathology, virology, bacteriology, parasitology, and screening of veins looking for gas emboli. Furthermore, samples for age determination, genetic studies, gastric content evaluation, stable isotopes and toxicology were taken from all the seven specimens.

The animals were part of the same group and determined by genetic and photo-identification to be part of the Mediterranean population. Causes of death did not include biological agents, or the “gas and fat embolic syndrome”, associated with direct sonar exposure. Environmental pollutant tissue concentrations were relatively high, in particular organochlorinated xenobiotics. Gastric content and morphologic tissue examinations showed a prolonged starvation, which likely caused, at its turn, the mobilization of lipophilic contaminants from the adipose tissue. Chemical compounds subsequently entered the blood circulation and may have impaired immune and nervous functions.

**Conclusions/Significance:**

A multi-factorial cause underlying this sperm whales' mass stranding is proposed herein based upon the results of *postmortem* investigations as well as of the detailed analyses of the geographical and historical background. The seven sperm whales took the same “wrong way” into the Adriatic Sea, a potentially dangerous trap for Mediterranean sperm whales. Seismic surveys should be also regarded as potential co-factors, even if no evidence of direct impact has been detected.

## Introduction

Sperm whale (*Physeter macrocephalus*, L 1758) mass strandings are mysterious events, which raise the concern and curiosity of the public opinion. The causes remain largely unknown, although many hypotheses have been considered and analyzed, including natural factors, such as biologic disease agents [1]; impairment of the navigation and echo-location systems due to bathymetric features, acoustic dead zones or anomalies of the Earth's geomagnetic field due to solar activity [2], [3], [4]; the effects of lunar cycles [5]; meteorological and oceanographic factors like local disturbances or basin-related temperature variations influencing prey distribution [6] and large-scale climatic events [7]. Furthermore, anthropogenic factors like noise pollution [8], [9] or environmental contaminants [10] have been also proposed as possible causes of strandings. A strong social component, which may prompt healthy animals to follow sick or disordered members of a pod, has been also considered as an additional relevant feature to be pondered in investigating the causes of mass strandings [11]. Mass mortalities involving sperm whales are usually clustered in determined geographical areas, such as the North Sea [1], [2], [3], [6], [8] and in the Southern Australian and New Zealand waters [4], [9], [10].

Although in the Mediterranean Sea the sperm whale is one of eight cetacean species considered to be regular inhabitants [12], mass strandings are rarely reported. In December 2009, a pod of seven sperm whales stranded along the coastline of the Gargano Promontory (Italy), in the Southern Adriatic Sea. Three animals were still alive and died within 48 hours after stranding. Sperm whales are considered to be vagrant or absent in the waters surrounding the stranding place, and particularly in the Central and Northern areas of the Adriatic Sea, where the habitat is not proper to this deep-diving species [13]. Sperm whales in the Mediterranean Sea occur preferentially in deep continental slope waters where mesopelagic cephalopods are most abundant [14], [15]. In fact, they have been frequently encountered in the Ionian Sea, especially along the Hellenic Trench [16], [17], [18], as in the Ligurian Sea, where they mostly occur along the continental slope, even if in these basins they were also reported in the pelagic areas [14].

In the Adriatic Sea, sperm whale mass strandings have occurred five times since historical times, with the oldest known instance dating back to 1584. In addition, some reports of single individuals stranded dead or alive included mention to one or more other sperm whales sighted at sea in the close proximity to the stranding location, sometimes for several days [15]. Groups stranded on the Adriatic Sea coasts (range 3–8 individuals) are smaller compared to the mean size of groups stranded outside of the Mediterranean Sea (range 18–30 individuals) [11], [19]. During this event, the Task Force for the Necropsies of Large Cetaceans, based at the Faculty of Veterinary Medicine of the University of Padua, funded by the Italian Ministry for the Environment, the Land and Sea, had the opportunity to coordinate an international and multidisciplinary research team throughout all *postmortem* investigations performed on these animals, thus giving rise, to the best of our knowledge, to the most exhaustive and comprehensive report concerning a sperm whale mass stranding.

## Results

**Figure 1:**
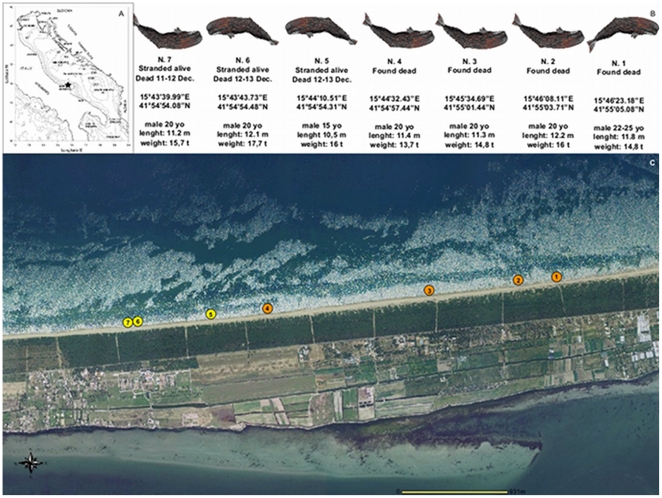
Site of the mass stranding event with main data of the seven stranded sperm whales. Fig. A (from the National Cartographic Portal) showed the exact location of the event along the Italian coastline and the bathimetric features of the Adriatic Sea; Fig. B and C detail main data and the position of the whales in respect to the shore.

On December 10 2009, the seven sperm whales were found stranded alive in shallow waters, parallel to the shore or beached, spread over a distance of 3,8 km on the Northern side of the Gargano Promontory (Fig. 1). The meteorological and the logistic conditions at the stranding site did not allow any refloating effort, so that the three animals died within 48 hours.

### Geographical and meteorological data

**Figure 2:**
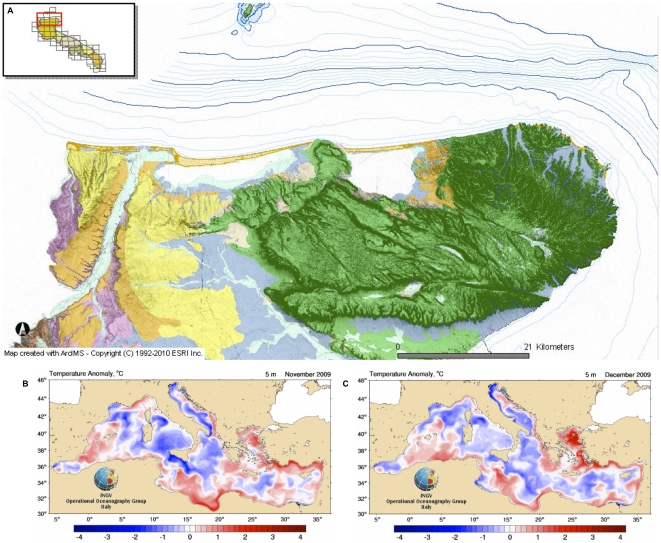
Hydrogeographical features of the stranding site and Superficial Sea Temperature (SST). Fig. A (from SIT-Regione Puglia) describes main hydrogeographical features of the stranding site; Fig. B and C report November and December average SST variation in the Mediterranean Sea compared to seasonal values.

A continuous long and narrow sandy coastline that faces the rather shallow central part of the basin, with a mean depth of 180 m, characterizes the vast majority the Italian side of the Adriatic Sea, including the stranding site. In general, the waters of the Adriatic more immediately adjacent to the Italian coasts possess a sandy seabed, which does not exceed 100 m of depth (Fig. 1 and 2A). The sea currents in the Adriatic Sea do not change dramatically, usually proceeding counter-clockwise going up along the Slavic and down along the Italian coastline. Marine currents usually enter the Adriatic Sea from the southern entrance of Otranto Strait and move towards the eastern Balcanic coast, turning westwards at different points (at the level of Bari, Pescara, Ravenna, and Venice).

Data obtained by international and local weather forecast and marine data archives showed strong winds (>7.5 m/s from 330°–350°N) blowing towards the shore, beginning the day before and persisting throughout the stranding day; anomalies of the marine currents (0.1–0.3 m/s faster than the local average) and high waves towards the Gargano Promontory were also reported, with a high tide at the time of stranding. In the month preceding the event, no relevant weather anomalies were described by the consulted reporting systems: normal disturbances and winds typical for the season interested both the Central and Southern Adriatic Sea, as well as the Ionian Sea. Furthermore, the trends of the currents did not show any significant change except on November 30, when all the currents in the Otranto Strait were directed northwards with strength greater than normal. A higher superficial temperature of the sea (2–4°C>than the seasonal average) was also recorded during November and December 2009 in the Hellenic Trench up to the entrance into the Adriatic Sea, as well as in the deepest part of the basin following the Eastern coastline (Fig. 2B and 2C).

The sun was at the minimum activity and no related changes of the geomagnetic field were reported during the month before the event studied herein. The moon was in the last quarter in the days of the stranding. Unusual natural events such as seaquakes, which have been proposed as a possible cause of stranding, were also investigated. In this respect, two events with a magnitude higher than 5 in the Richter Scale were considered: a quake on November 3 (ID 2209537880, magnitude 5.9 in the Ionian Sea in front of Zakynthos, GR) and another similar event on November 26 (ID 2209874680, magnitude 5.1 in the Southern Hellenic Trench, about 150 km from the Peloponnese coastline).

### Biological data, genetic and stable isotope analyses

The seven male sperm whales were 10.5 to 12.2 m long, with an estimated age between 15 and 25 years, calculated by counting dentin growth layer groups (GLG). Biometrical data regarding the seven animals are summarized in Table 1. A comparison between expected and corrected body weights, the first ones estimated using the animals' total length, the latter ones determined considering also *postmortem* body fluid and tissue leakages, suggested a moderate weight loss (with an average loss of 2.15 tons per animal).

**Table 1:** Biometrical data and age determination of the 7 stranded sperm whales.

Sperm whales	no. 1	no. 2	no. 3	no. 4	no. 5	no. 6	no. 7
**Total length (m)**	11,8	12,2	11,3	11,4	10,5	12,1	11,2
**Extimated weight (t)**	18,9	20,7	16,7	17,1	13,7	20,4	16,3
**Measured weight (t)**	13,0	14,0	12,5	12,0	14,0	15,5	13,8
**Corrected weight (t)**	14,8	16,0	14,8	13,7	16	17,7	15,7
**Age estimation (y.o.)**	25	20	20	20	15	20	20

Preliminary analyses of population genetic structure revealed that all the stranded whales, similarly to other ones sampled within the Mediterranean Basin, belonged to a unique haplotype, with no genetic variability at the mitochondrial level (*h* = 0.00, π = 0.00).

Using photo-identification methods, three of the seven individuals matched with previously observed free ranging sperm whales (*Cla*, *Pomo*, *Zak Whitehead*). The whale named *Cla* (no. 6) was a male photographically captured for the first time in 2002 in the Western Ligurian Sea, where it was re-captured five more times in 2003, 2005 and 2007. It was always observed as a solitary individual, or among males. The whale named *Pomo* (no. 2) was a male photographically recorded for the first time in 2003 in the Western Ligurian Sea in a male association pod. The whale named *Zak Whitehead* (no. 5) was a male that was first photographically captured in 2000 in the Southeastern Ionian Sea, in the so-called “Hellenic Trench”, and here re-captured seven times in 2002 and 2005. It was always observed as member of the same social unit (*sensu* Whitehead 2003; group of adult females, immature whales and occasionally calves with a more or less stable composition).

Stable isotope analyses revealed an expected average for δ^13^C (−18.5±1.2‰), while δ^15^N showed on average fairly low values (9.8±0.4‰). A higher isotopic variability was found between tissues (on average Δ = 2.8‰ and 0.8‰ for δ^13^C and δ^15^N, respectively) than between specimens (on average Δ = 1.4‰ and 0.4‰ for δ^13^C and δ^15^N, respectively). Blubber was the most ^13^C-depleted (−19.5±0.9‰) and ^15^N-enriched body tissue (10.2±0.1‰), while muscle was the most ^13^C-enriched (−16.7±0.5‰) and liver the most ^15^N-depleted (9.3±0.3‰) tissue.

### Pathological and other findings

**Figure 3:**
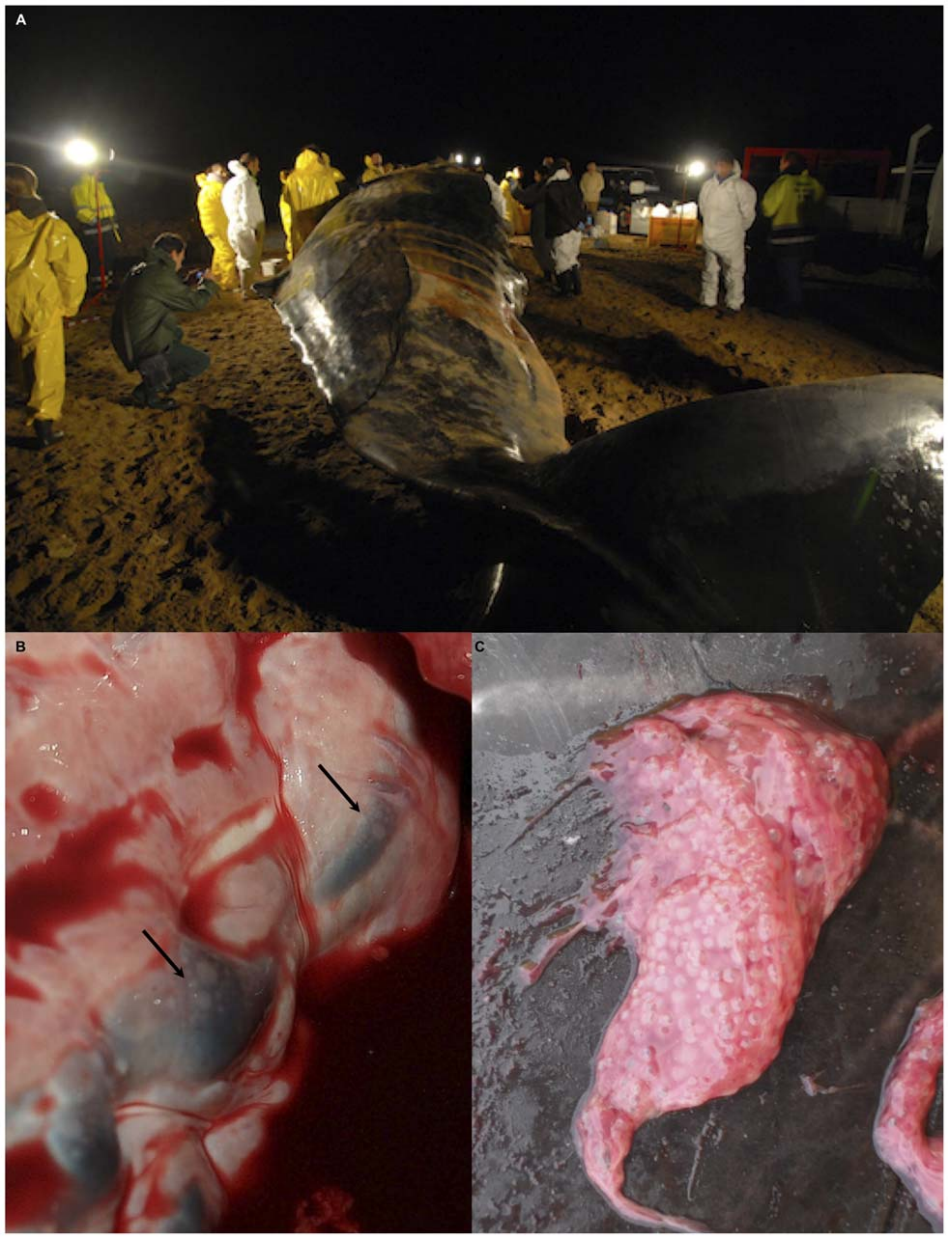
Nocturnal necropsy and gas bubbles. Fig. A shows a scene of the nocturnal necropsy on animal no. 7. In Fig. B and C gas bubbles in the heart veins (black arrows) and in an intracardiac clot of animal no. 5 are shown.

As stated before, for the working conditions, detailed *postmortem* examinations were thoroughly carried out on the three sperm whales that stranded alive, requiring an average of 9 consecutive hours for each whale (Fig. 3A). On the other hand, the progressively advanced cadaveric changes affecting the carcasses of the other four animals, all found stranded lifeless, limited our investigations to an external inspection, followed by a careful evaluation of the gastric contents.

#### a. Gross lesions

A mild cutaneous parasitic infestation was observed at external examination: specimens of copepods belonging to the genus *Pennella* (Siphonostomatoidea: Pennellidae) were found embedded within the host blubber of all seven animals, often associated to a granulomatous or purulent inflammatory reaction around chitinous remains of the parasites, sometimes accompanied by opportunistic agents (bacteria and fungi), as clearly shown by subsequent histopathological investigations. Tetraphyllidean larvae (Cestoda) were found a) in the subcutaneous blubber of one sperm whale and identified as *Phyllobothrium delphini*; and b) in the mesenteries of all the examined animals and classified as *Monorygma grimaldii*. Examining the abdominal cavity of the three studied animals, a mild, chronic, fibrino-erosive peritonitis was also observed, with the thoracic cavity showing a more or less evident pneumothorax associated with a severe pneumomediastinum and with the presence of empty cavities involving the inner cortex of pulmonary lymph nodes, surrounded by hemorrhages. Numerous gas bubbles, shaped as trains or clusters, could be observed in the coronary veins of the three sperm whales (Fig. 3B). Scarce small bubbles were observed only in subcutaneous veins of sperm whales no. 5 and 6 and in mesenteric veins of no. 5. Bubbles were also found entrapped into a large intra-cardiac blood clot in the latter sperm whale (Fig. 3C). All the remaining veins were congested, without evidence of bubbles. We emphasize that concurrent fat embolism was ruled out since all the sections from the lungs of the three sperm whales examined showed no or very limited histochemical evidence of lipid droplets in pulmonary vessels, with no clinic or pathological significance. Petechiae and bruises were frequently encountered in the lungs, pleura, mesenteric lymph nodes, and in the kidneys, with a severe congestion of all the tissues mentioned above. Prescapular and pulmonary lymph nodes were markedly enlarged and edematous, with a chronic reactivity being also evident on their cut surfaces, associated with the presence of hemorrhagic foci and brown to black discolored areas.

#### b. Gastric contents

Stomach contents consisted chiefly of highly digested cephalopod beaks and foreign bodies. Both the organic and inorganic fractions occurred in all the seven animals, although the gastric chambers were almost empty in individuals no. 4 and no. 5. The intestines of all the three whales examined at necropsy were empty; the scarce content of the lumen was due to an abundant, biliary-stained mucous-like content. In all the animals that we examined we noted no fragments of cephalopod flesh, gladii (entire or partial), sucker rings, hooks or eye lenses; beaks were found in varying stages of digestion and no fish or crustaceans remains were observed. On the basis of the morphological features of the squid beaks, members of the histioteuthid family were the commonest prey, specifically *Histiotheutis bonnellii* and, to a lesser extent, *H. reversi* and other rare species (*Octopoteuthis sicula* and *Galiteuthis armata*). Gastric nematodes, which were present in almost all samples, represented the second part of the undigested material. Several adults and pre-adults of *Anisakis* spp. (Nematoda: Anisakidae) were observed in the stomach chambers in association with a mild, multifocal, catarrhal gastritis. Table 2 summarizes the gastric findings and shows how parasitic burden increased with the amount of both fractions, organic and inorganic. Inorganic remains comprised mostly foreign bodies of human origin, including fishing gear and hooks, ropes, and several plastic objects. No evident obstruction or perforation of the alimentary tract was noted.

**Table 2:** Gastric contents of the 7 stranded sperm whales.

Sperm whales	no. 1	no. 2	no. 3	no. 4	no. 5	no. 6	no. 7
**Organic fraction weight (g)**	208,9	n.e.	237,9	10,6	29	496,2	265,4
**n. Anisakis spp.**	+	n.e.	+	0	41	244	145
**Inorganic fraction weight (g)**	1475,4	n.e.	4934,6	20,7	9,5	1199	731,6

- +: present but not quantified.

- n.e.: not evaluated.

#### c. Histopathology

**Figure 4:**
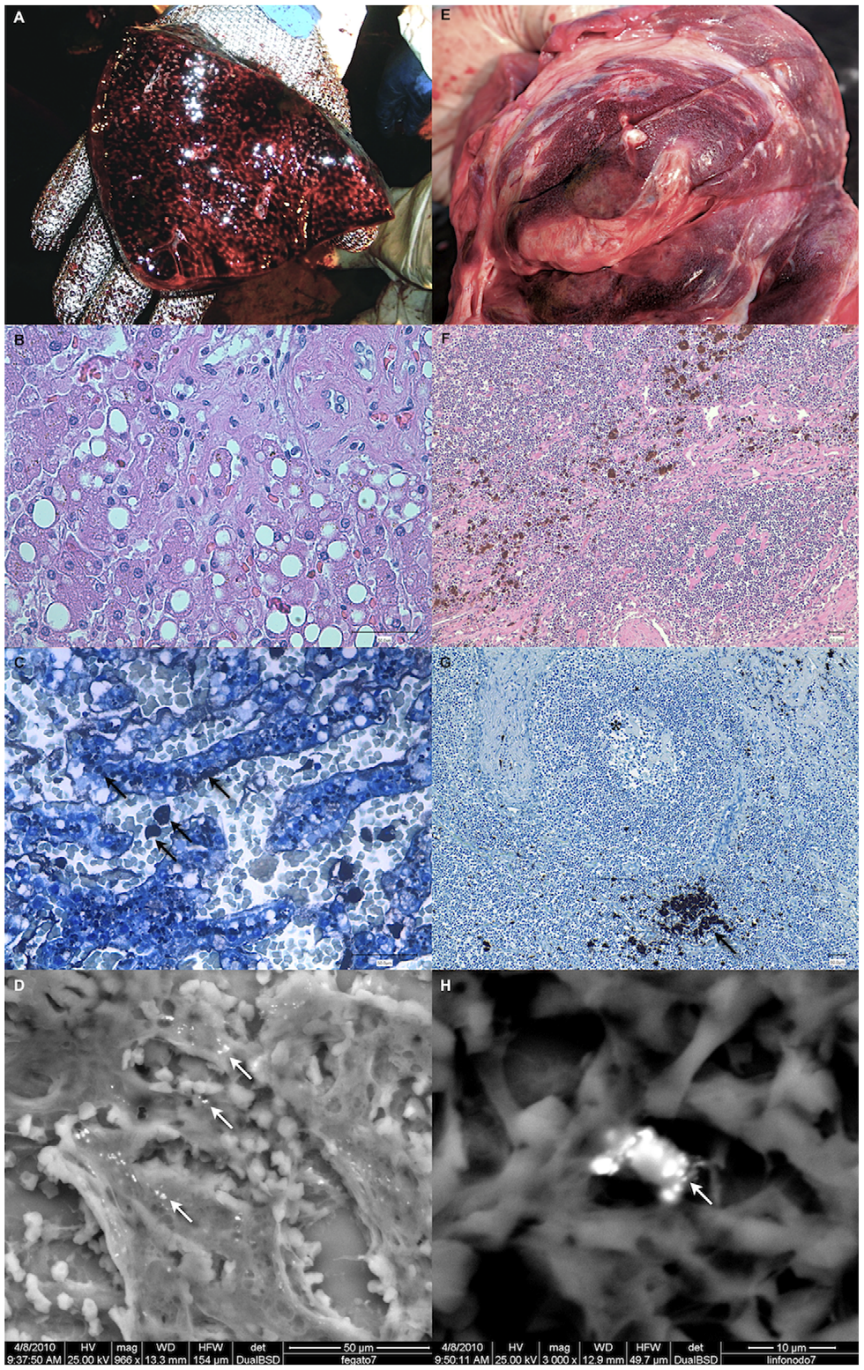
Liver and lymph node pathological changes related to Hg presence. Gross features, hematoxylin and eosin (HE) and automethallographic staining, along with ESEM pictures of liver (respectively, Fig. A–D; Fig. B and C have a 10× magnification) and lymph nodes (respectively, Fig. E–H; Fig. B and C have a 40× magnification). In HE-stained sections, hepatic macrovacuolar lipidosis in the liver and macrophages loaded with brownish pigments are evident. HgSe crystals can be observed in the liver (hepatocytes and Kuppfer cells) and lymph nodes (sinusal macrophages) both with the automethallographic staining (black arrows) and ESEM pictures (white arrows). In Fig. G also follicular lymphoid depletion is prominent (_*_).

An acute, multifocal to diffuse, fibrinous bronchopneumonia and a mild, diffuse, chronic catarrhal enteritis were microscopically observed in all the three animals examined at necropsy. Histological investigations confirmed also the severe hyperemic and hemorrhagic lesions observed during gross examinations. In particular, the liver exhibited a severe diffuse congestion of sinusoids with prominent compression of hepatocytes, accompanied by a multifocal macro-vacuolar steatosis that was prominent around portal triads. Lymph nodes were histologically characterized by a severe and diffuse intra-follicular lymphoid cell depletion, associated with the deposition of a hyaline eosinophilic amorphous material at the cortical level, while a diffuse and severe histiocytic infiltration was observed in sinusal spaces. Several solitary or aggregated macrophages showed a brown to black cytoplasmic pigmentation, which positively stained with the auto-metallographic technique (Danscher's staining), a specific histochemical staining for inorganic mercury. In general, the above mononucleated elements had mostly perifollicular and perivascular locations. By means of the auto-metallographic technique, we confirmed the presence of inorganic mercury also in the liver and kidneys of all the three whales examined with a full necropsy, as well as in brainstem neurons of animal no. 6. In the liver, Hg was restricted to the cytoplasm of hepatocytes and Kuppfer cells and especially concentrated in periportal areas, whereas in the kidney Hg was confined to the cytoplasm of tubular epithelial cells, with special reference to those of cortical tubules. At the same time, the aforementioned cytoplasmic granules detected in neurons, hepatocytes, Kuppfer cells, and macrophages of the lymph nodes, stained positively also with the Schmorl's histochemical technique commonly used to mark lipofuscin pigments. Only in myocardial and musculoskeletal fibers lipofuscin granules were observed despite the absence of inorganic mercury particles. The observation of the aforementioned cytoplasmic crystals by environmental scanning electron microscopy (ESEM), coupled with x-ray induced fluorescence, confirmed a composition similar to mercury selenide (HgSe, composed for 71.25% of total weight by Hg and 28.25% of total weight by Se): hepatic granules were composed by Hg (71% of total weight), Se (24.37% of total weight), and S (4.62% of total weight); granules in the macrophages of lymph nodes were constituted by the same elements, albeit with slightly different proportions (Hg 73.44%, Se 20.22% and S 6.34% of total weight). Hepatic and lymph node findings are shown in Fig. 4.

#### d. Microbiological investigations

The extensive microbiological investigations performed on selected organs from the three fully examined whales revealed the presence of opportunistic bacteria (*Vibrio* spp., *Aeromonas hydrophila*, and *Enterococcus* spp.) in lungs, pulmonary lymph nodes, intestine and kidneys, sometimes associated with acute inflammatory changes, mainly in the respiratory tract.

#### e. Other diagnostic tests

*Brucella* spp was not detected in the same whales, similarly to a number of viral agents, which have been reported to be the causative agents of several mortality episodes among free-ranging cetaceans (*Morbillivirus*, *Herpesvirus*, *Coronavirus* and *Adenovirus*), searched using both bio-molecular techniques and electron microscopy. Analyses for *Toxoplasma gondii* gave positive results in animals no. 6 (mesenteric, prescapular lymph nodes, and pancreas) and no. 7 (prescapular lymph nodes and liver), with no evident associated microscopic changes. All DNA extracts scored positive upon *T. gondii*-specific PCR: the B1 sequences generated were 95 bp long and identical to each other, displaying 100% nucleotidic homology with the B1 sequence deposited in the GenBank™ (Accession Number AF179871), thus confirming that these two whales harbored *T. gondii* in their tissues.

### Toxicology, biotoxins and biological trials

#### a. Trace metals

**Figure 5:**
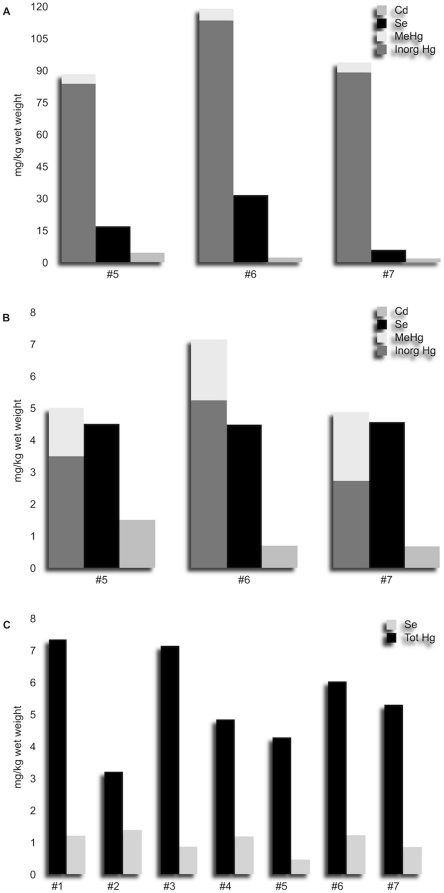
Hg, Se and Cd concentrations in sperm whale tissues. Graphics summarize tissue Hg (inorganic and organic), Se and Cd concentrations in liver (Fig. A), and kidneys (Fig. B) in the three necropsied whales and total Hg and Se in muscular tissue of all the seven animals (Fig. C).

The total hepatic and renal Hg concentrations and the liver organic Hg fraction were quite similar within the group (Table 3). Conversely, differences were noted in methyl-mercury percentages in tissues from the three animals examined at necropsy, with the renal fraction of the total Hg load (26.6%, 30.14% and 44.06%) being relatively higher than in other tissues (Table 4). MeHg mostly represented the content of Hg in muscles. Heavy metal analyses on muscles sampled from all the seven whales confirmed that the trace metal profile was similar for the entire pod. Se concentrations in the examined tissues were relatively high: in the livers of the three examined animals the molar Hg∶Se ratio was close to 1∶1, while in the other two examined tissues, in particular in kidneys, higher concentrations of Se were detected. High levels of Cd were observed in renal tissue. Similar patterns of heavy metal concentration and ratios were observed in the muscles of the entire pod (Table 5). Trace element tissue concentrations are represented in Fig. 5.

**Table 3:** Heavy metals (Hg, Se, Cd; mg/kg w.w.) and POPs (HCB, PCBs, DDTs, Total and Carcinogenic PAH; ng/g lwt.) concentrations in livers of the three necropsied sperm whales (no. 5, 6 and 7).

Sperm whale	no. 5	no. 6	no. 7
**TotHg (mg/kg ww)**	88,3	119	93,8
**MeHg (mg/kg ww)**	4,46	5,52	4,67
**%MeHg**	5,05	4,64	4,98
**Se (mg/kg ww)**	17,03	31,66	6,01
**Hg∶Se (molar)**	1,43	0,87	1,33
**Cd (mg/kg ww)**	1,51	0,70	0,69
**HCB (ng/g lwt)**	51,94	246,75	246,38
**DDTs (ng/g lwt)**	9075,33	139290,00	49032,71
**PCBs (ng/g lwt)**	7134,81	135223,7	50896,19
**Tot PAHs (ng/g lwt)**	891,84	796,16	960,7
**Carc PAHs (ng/g lwt)**	194,94	169,89	80,77

**Table 4:** Heavy metals (Hg, Se, Cd; mg/kg w.w.) concentrations in kidneys of the three necropsied sperm whales (no. 5, 6 and 7).

Sperm whale	no. 5	no. 6	no. 7
**TotHg (mg/kg ww)**	5,01	7,15	4,88
**MeHg (mg/kg ww)**	1,51	1,90	2,15
**%MeHg**	30,14	26,57	44,06
**Se (mg/kg ww)**	4,51	4,49	4,58
**Hg∶Se (molar)**	0,47	0,51	0,44
**Cd (mg/kg ww)**	4,61	2,34	1,91

**Table 5:** Heavy metals (Hg, Se, Cd; mg/kg ww) and POPs (HCB, PCBs, DDTs, Total and Carcinogenic PAH; ng/lwt) concentrations in the muscular tissue of the seven sperm whales.

Sperm whale	no. 1	no. 2	no. 3	no. 4	no. 5	no. 6	no. 7
**TotHg (mg/kg ww)**	7,35	3,22	7,15	4,85	2,98	7,03	5,16
**Se (mg/kg ww)**	1,21	1,39	0,87	1,19	0,47	1,23	0,86
**Hg∶Se (molar)**	2,38	0,91	3,25	1,60	2,50	2,25	2,36
**Cd (mg/kg ww)**	0,008	0,006	0,004	0,006	0,006	0,003	0,004
**HCB (ng/g lwt)**	33,69	98,26	89,28	93,66	66,47	156,16	18,69
**DDTs (ng/g lwt)**	13899,66	19988,41	27597,67	21305,98	11352,15	122086,30	3907,08
**PCBs (ng/g lwt)**	12148,43	25162,86	29477,59	20754,51	9488,29	104106,6	4320,10
**Tot PAHs (ng/g lwt)**	3747,43	513,41	1395,84	1339,47	1199,86	3444,95	1195,65
**Carc PAHs (ng/g lwt)**	144,90	67,17	49,16	28,13	32,22	128,51	31,74

#### b. Organochlorines

**Figure 6:**
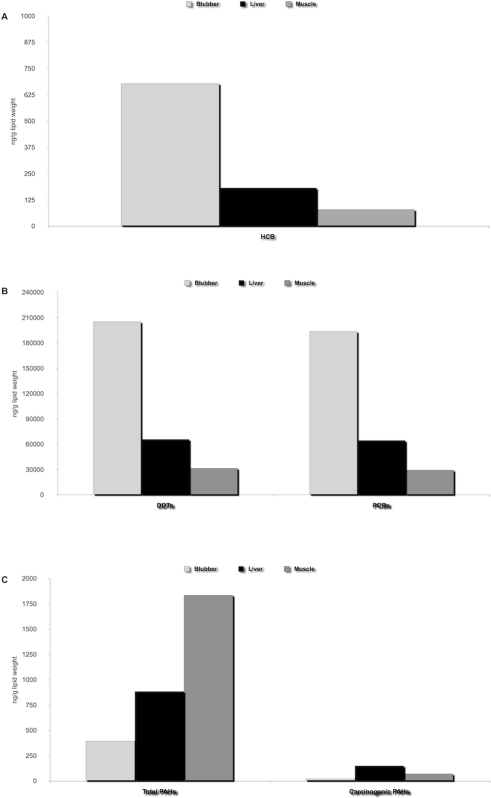
HCB, DDTs, PCBs and PAH concentrations in sperm whale tissues. Figures compare HCB (A), DDTs and PCBs (B), as well as Total and Carcinogenic PAHs (C) concentrations in blubber, liver and muscle of the seven specimens of sperm whales.

Analyses on organic pollutants showed that total DDTs were the most represented class of organochlorinated compounds, despite a >40 years old international ban. Total polychlorinated biphenyl (PCB) levels were also significantly high in all the whales, while hexachlorobenzene was the least represented compound. All organochlorinated classes showed a similar distribution with the higher quantity being measured in the blubber, followed by the liver and muscles tissues. The hepatic, muscular and blubber concentrations of HCB, DDTs and PCBs are reported in Tables 3, 5, and 6. The statistical analysis using the non-parametric test of Kruskal-Wallis to compare the levels of the three considered xenobiotics, confirmed significant differences (p<0.025) between the three biological matrixes for HCB (Fig. 6A) and DDTs (Fig. 6B), but not for PCBs (Fig. 6B). In particular, these differences are significant (p<0.025) comparing blubber and muscle data for both the organochlorinated compounds using Kolmogorov-Smirnov test.

**Table 6:** POPs (HCB, PCBs, DDTs, Total and Carcinogenic PAH; ng/lwt.) concentrations in the blubber of the seven sperm whales.

Sperm whale	no. 1	no. 2	no. 3	no. 4	no. 5	no. 6	no. 7
**HCB (ng/g lwt)**	107,74	191,26	244,77	3650,15	130,7	23.891	179,62
**DDTs (ng/g lwt)**	38890,64	43104,51	84966,67	948049,9	29166,01	259549,2	32507,49
**PCBs (ng/g lwt)**	24628,99	45799,79	74656,54	950852,00	19668,76	206002,9	33629,46
**Tot PAHs (ng/g lwt)**	339,02	276,15	552,46	244,33	310,06	136,47	446,73
**Carc PAHs (ng/g lwt)**	28,91	15,90	13,48	19,07	19,70	15,53	32,98

#### c. Polycyclic aromatic hydrocarbons

Polycyclic aromatic hydrocarbons (PAHs) were low but showed a peculiar distribution as compared to organochlorinated xenobiotics, with higher mean values in musculoskeletal tissues, followed by liver and then blubber (Fig. 6C). The hepatic, muscular and blubber concentrations of PAHs are reported in Tables 3, 5, and 6. The statistical analysis using the non-parametric test of Kruskal-Wallis confirmed significant differences (p<0.025) between liver, muscles and blubber for total and carcinogenic PAHs. Kolmogorov-Smirnov test showed that total and carcinogenic PAH adipose tissue levels were significantly lower (p<0.025) than in liver and in muscle, while the amount of PAHs in liver was significantly lower only for total ones (p<0.10).

#### d. Biotoxins

The analyses for hydrophilic and lipophilic biotoxins were negative.

#### e. Other compounds

Neither organophosphates nor carbammates were found in sperm whale tissues.

#### f. Biological trials

The mosquito fishes exposed to sperm whales' liver extracts showed an abnormal swimming pattern in the first hours when compared to the animals exposed to porcine livers. A mild inhibition of acetyl-cholinesterase enzyme (about 35% in comparison to the control group) was assessed after 48 hrs from exposure, supporting the hypothesis of the presence of compounds with neurotoxic effects in the sperm whales' livers.

## Discussion

Mass strandings of sperm whales are extremely rare in the Mediterranean Sea. In the Adriatic Sea, five similar events involving 3–8 animals were reported since 1555 before the one considered in this study [15], even if this species is regarded as opportunistic or vagrant in the basin [12]. In the present sixth instance, occurred on December 10–11, 2009, the 7 animals came in from both Western and Eastern Mediterranean basins. The comparative analyses of the entire control region of the mitochondrial DNA confirmed that the seven whales belonged to the Mediterranean subpopulation. The total lack of haplotype diversity among samples collected within the Mediterranean Sea implies some degree of population isolation, small effective population size or perhaps a reduction in maternal lineages brought on by a recent bottleneck event [20], [21]. The low level of haplotype variability found in this investigation was also reported in previous studies [22]. These latter investigations have indicated substantially low levels of nucleotide variation on a global scale along with the presence of significant levels of kinship between some group members that may be the result of matrilineal structuring at the unit or group level [20], [23], [24], [25]. Lyrholm et al. (1998) proposed a historical bottleneck to explain the low levels of mtDNA diversity (based on the data obtained on a DNA 320bp sequence from the control region) [23]. Studies on stable isotopes performed on target tissues revealed that the average δ^13^C was within the literature range for sperm whales over various geographical areas [26], [27], with low δ^15^N levels. This finding mirrors the oligotrophic conditions of the Mediterranean Sea [28], [29], thus confirming the Mediterranean origin of the sperm whales. The hypothesis that they belonged to the same pod was put forward based on the genetic outcomes, which identified the mitochondrial control region sequence to be identical for all stranded animals. The low between-specimen isotopic variability indicated also similar dietary sources [30], [31]. In any case, the exact type of association among these whales before the stranding remains unknown, although their number is larger than the group size of loose male aggregations in the Mediterranean [19], [32], [33].

Stable isotope analysis was used also to investigate dietary history and feeding levels as well as to understand any differences in the timing of movements [26], [31]. The tissue isotopic variability observed in this study can account for differences in short- and long-term diet, due to a different cellular turnover. To our knowledge, no specific information on sperm whale tissue turnover is available. However, skin turnover is considered comparable to that of other odontocete species (about 70–75 d) [32] and its isotopic composition thus provides dietary insight over periods of more than 2 months. Other tissues, and especially muscle, are expected to have a longer turnover, thus providing long-time integrated information on diet. Since sperm whales are known to have a wide habitat range on the offshore part of the continental slope, albeit having a very specialized diet on cephalopods [34], the isotopic differences between short- (e.g. skin) and long-turnover tissues (e.g. muscle) suggest the exploitation of different feeding areas. The factors expected to affect the behavior of the seven sperm whales under study, and possibly causing their stranding, arose about 10 to 20 days before the event: therefore, the temporal resolution of stable isotopes does not allow to gain specific insights into very recent movements, and the possible concurrent role of starvation in this mass stranding event cannot be ruled out. Despite the remaining gaps and the several still open issues, our data suggest migration of sperm whales from one deep Mediterranean basin to the other either through the Strait of Messina or through the Strait of Sicily. In this respect, the numerous single sperm whales' reports obtained by the National Stranding Database are mainly around the first way suggesting that the Strait of Messina could be likely the preferred migration route.

Despite all these observations, we were not able to confirm that these stranded sperm whales formed a single stable group with a social hierarchy, although we would rather suggest that more than one loose male aggregation and/or several solitary individuals could have coalesced in a limited sea area, most likely in the Ionian Sea, between summer and fall. From there they subsequently entered the Adriatic Sea for unknown reasons. To the best of our knowledge, no relevant unusual natural events (i.e. seaquake or weather storms) or noxious anthropogenic activities (military drills using sonar) that could have caused an avoidance behavior occurred temporally and spatially associated with the event. The only relevant anomaly reported by the marine data archives was the increased sea superficial temperature in November and December along the Hellenic Trench and Eastern part of the Adriatic Sea, possibly constituting a thermal front in which upwelling and/or downwelling could have been favorable to the development of cephalopod populations. Several studies have documented the influence of frontal zones on sperm whale distribution worldwide. This species and other teutophageous cetaceans (i.e. dwarf and pigmy sperm whales, pilot whales, Risso's dolphin, and Ziphiidae) occurred, over abyssal depths, at the steepest sea superficial temperature gradients, at the periphery of a cyclone zone and in a convergence zone, both forming thermal fronts because of the aggregation of main preys near these zones [34], [35].

The “Hellenic Trench”, the likely winter aggregation area, is 600 km (Lefkada Island) to 1100 km (Crete) away from the stranding site (distance calculated on a straight way with no deviation due to marine currents). Considering the maximum horizontal speed reported for male sperm whales (90 km/day) [11], with no slowdown due to feeding activities or weakness, it took no less than 7 days for these whales to reach the Gargano Promontory. In this respect, the low quantities of highly digested squid beaks found within the gastric cavities are in open contrast with the feeding habits and daily intake typical of the species [36], thus suggesting a starvation period of at least 3 to 7 days [11], [36], an amount of time compatible with the traveling time. Furthermore, the mild portal hepatic steatosis observed at microscopic examination, along with the real body weights of the seven animals (calculated using a correction factor applied to the weight measured during disposal of the carcasses), that were lower than the expected values (estimated on the basis of the total body length), further support this hypothesis [1]. Foreign bodies (including fishing gears and hooks, ropes, and plastic objects) were found in all the examined stomachs, with an incidence higher than those reported for other mass strandings [36]. Nevertheless, all the objects recovered from the whale stomachs cannot be proposed as a likely cause of stranding, given the absence of any evident obstructions [37].

The mass stranding that we report and discuss here occurred about 200 km South of the Italian Adriatic sandy coastline between the 45° and the 42°30′ parallel, on which most of the historical stranding occurred [15]. All these areas display geographical features similar to those defined “acoustic dead zones” [4]: the comparable morphology of the stranding location and the meteorological conditions registered during the days before the event (winds, currents and waves directed to the Gargano coasts) could explain why our seven sperm whales arrived on their stranding and beaching destination. Preliminary observations, in particular the distribution and the position related to the coastline, suggested that all animals were debilitated, possibly by a common pathological condition. We emphasize that the presence of copepods of the genus *Pennella*, that affected the skin of the seven whales, has been suggested as a reliable indicator of poor health in free-ranging cetacean populations [38].

The field working conditions limited the analysis to those animals found agonizing on the shore that died in the following days. In fact, many of the observed pathological findings (i.e. diffuse visceral congestion and hemorrhages; mild fibrinous peritonitis; pneumothorax) were likely related to recumbence. The search for infectious agents and marine biotoxins responsible for known cetacean epidemic outbreaks and mass die-offs was negative [39], [40]. However, an impairment of the immune response was strongly suspected on the basis of the prominent lymphoid cell depletion that was apparent in several lymph nodes, as well as on the basis of both the acute opportunistic bacterial infections ascertained in the respiratory tract and of the biomolecular demonstration of *T. gondii* within a wide range of tissues sampled from the three whales on which a detailed necropsy was performed. As a matter of fact, and similarly to what reported in terrestrial mammals, *T. gondii* is commonly believed to be an opportunistic protozoan agent in free-ranging cetaceans [39]. Since no infections due to a lymphotropic agent (i.e. *Morbillivirus*) were microscopically, molecularly and serologically detected on *postmortem* examination, the primary cause of lymphoid depletion remains uncertain. Bacterial components (such as lipopolysaccharides), prolonged stressful conditions, malnutrition and emaciation observed in diseased whales, might all contribute to immune system impairment [41], [42]. In the whales examined in this study, the acute fibrinous pneumonia caused by opportunistic bacteria, along with the biomolecular identification of *T. gondii* in two animals, should be regarded as very reliable biologic indicators of a pre-existing damage of the immune response. In this respect, the lack of pathological *T. gondii*-associated changes in all tissues examined supports the hypothesis of a recently acquired infection. In our opinion, both the high tissue levels of environmental pollutants and the prolonged starvation period should be considered as the most likely causes of the whales' immune response impairment. Previous studies on harbor porpoises (*Phocoena phocoena*) confirmed the existence of a relationship, in odontocete cetaceans, between lymphoid cell depletion in lymphatic tissues and elevated persistent organic pollutant body burdens, thus strongly supporting the hypothesis of a contaminant-induced failure of the immune system [42], [43].

The direct impact of acoustic sources (sonar or air-guns for seismic surveys) or a seaquake that could have caused the onset of the “gas and fat embolic syndrome” was considered as another possible cause of death [44]. Nevertheless, this suspicion was ruled out, since the three fully examined whales showed no evidences of lipid and gas emboli. Interestingly enough, our results concerning pulmonary fat emboli confirmed that “gas and fat embolic syndrome” is not commonly observed in stranded cetaceans, even if they spent hours stranded alive on the shores, thus clearly differentiating the above condition from atypical mass stranding related to sonar exposure [44]. On the other hand, gas bubbles were mainly found in the coronary veins and not widely distributed throughout the rest of the body as described in the “gas and fat embolic syndrome”. Two hypotheses could be proposed to explain the presence of gas bubbles in the coronary veins. The first one points to the prolonged lateral recumbence of these very large and heavy whales on the beach, with almost or no chance of thoracic movement that progressively led to over-breathing with overexpansion of the passively congested lungs and rupture of alveolar walls, followed by interstitial and sub-pleural emphysema, as confirmed by thoracic hemorrhages, leading to pneumomediastinum, likely pneumothorax, and pneumopericardium [45]. This process can result in gas entering the pulmonary veins and lymphatic vessels, thus reaching either the left atrium and left ventricle, or the lymph nodes, respectively. This process reduces pulmonary ventilatory efficiency and leads progressively to general hypoxia and cardio-pulmonary hypertension and failure. The second option suggests that the large and unexpected journey of these deep diving whales through shallow waters could enable free gas offloading from tissues, worsened by stranding. Congested lungs hamper bubbles' clearance at the same time as gas offloading is promoted by a low ambient pressure (surface). Finally, both processes may simultaneously occur and synergistically influence each other.

We have no information of any naval seismic surveys being performed at the time of stranding or during the preceding weeks. Notwithstanding this, the Italian Government released several authorizations for such surveys for the Central and Southern Adriatic Sea. In the present study, we obtained no evidence of those pathological changes and mild behavioral abnormalities, which have been spatially and temporally linked to seismic experimentations and described in sperm whales from the Gulf of Mexico [46]. Nevertheless, acoustic trauma and consequent disorientation of the whales cannot be fully ruled out as concurrent causes of the mass stranding reported herein.

The total hepatic and renal mercury concentrations and the liver organic Hg fraction were similar to those previously found in other odontocetes in the Mediterranean Sea [47], [48], although higher than those measured in sperm whales involved in other mass strandings [49]. A circulation of methyl-mercury in tissues of the three whales that underwent a full necropsy was hypothesized, based on its concentrations in kidneys and relative percentages on total Hg. The circulation of methyl-mercury originating from body storages has been confirmed in fasting or lactating laboratory animals, in which organic mercury enters the blood circle and subsequently spreads to all main target organs, including the kidneys [50]. In addition, low protein diet feeding, in particular with low sulfur amino-acids, associated with low organic mercury concentrations, are suspected to decrease urinary Hg excretion and simultaneously increase the retention of MeHg metabolite in renal cells, by a more efficient re-adsorption of MeHg than in normal protein diets and modulation of the activity of glutathione complex and thiols [51], [52]. The deposition of inorganic mercury as black auto-metallograpic positive granules within the cytoplasm of macrophages residing in the enlarged, brown to black-pigmented prescapular lymph nodes, as well in hepatic Kupffer cells, does also suggest a circulation of inorganic mercury. In this respect, the ultrastructural investigations carried out on the liver samples and the Hg∶Se close to one ratio found with trace element analysis confirmed that in sperm whales, as in other odontocetes, hepatic organic mercury precipitates into tiemannite (HgSe) crystals as an end product of methyl-mercury detoxification [53], [54]. Any possible degenerative changes, included those prompted by prolonged fasting, are believed to cause the release of cytoplasmic HgSe complexes into the bloodstream, with subsequent macrophage phagocytosis. This observation supports the hypothesis that in cetaceans mononuclear phagocytes are crucial for the biological fate of Hg [55]. *In vivo* studies on mice have also demonstrated that a selective increase of the inorganic Hg fraction in lymph nodes is likely due to the demethylation process of MeHg that takes place in macrophage-rich lymphoid tissues. A similar mechanism could determine, at its turn, a preferential accumulation of inorganic Hg within the lymphoid tissues in comparison to other non-lymphatic body districts, following exposure to MeHg [56]. Cadmium concentrations in the kidney and other tissues showed levels similar to those observed in marine mammals and birds with a diet based on cephalopods [57].

Furthermore, our toxicological investigations on the main persistent organic pollutants (POPs) demonstrated increased levels of PCBs and DDT metabolites in tissue samples from of all the seven whales, as well as in the liver from the three individuals that underwent a full necropsy. Tissue levels of polycyclic aromatic hydrocarbons (PAHs) were low. However, their muscular concentrations higher than those measured in blubber and hepatic tissues, may suggest a release of such compounds into the blood circulation from body lipid reserves, since the affinity of PAHs for fat tissues is lower than that of other POPs. Organochlorinated compound levels, in particular PCBs and DDTs, were higher than those found in other sperm whale mass strandings [10], [49], as for the liver concentrations of Hg. The presumed circulation of organochlorinated pollutants in association with mercury was likely due to the prolonged fasting period [58], [59]. The synergistic interaction between these two types of contaminants possibly enhanced their neurotoxic effects [60], [61], [62].

To summarize, we believe that a group of sperm whales, not familiar with the South Adriatic marine area, entered the basin for feeding strategies, or to avoid one or more concurrent human or natural disturbances, or because of other hitherto unknown reasons. The cetaceans swam northward toward a dead end and soon found themselves starving. Prolonged starvation, environmental conditions improper for the species, along with breakdown of adipose body reserves and the consequent release into the bloodstream of chemical substances likely displaying neurotoxic and immunotoxic effects, altered the orientation and space perception of the whales, worsening their welfare and health. Prevailing meteorological conditions finally led the cetaceans to strand on the Gargano Promontory.

**Figure 7:**
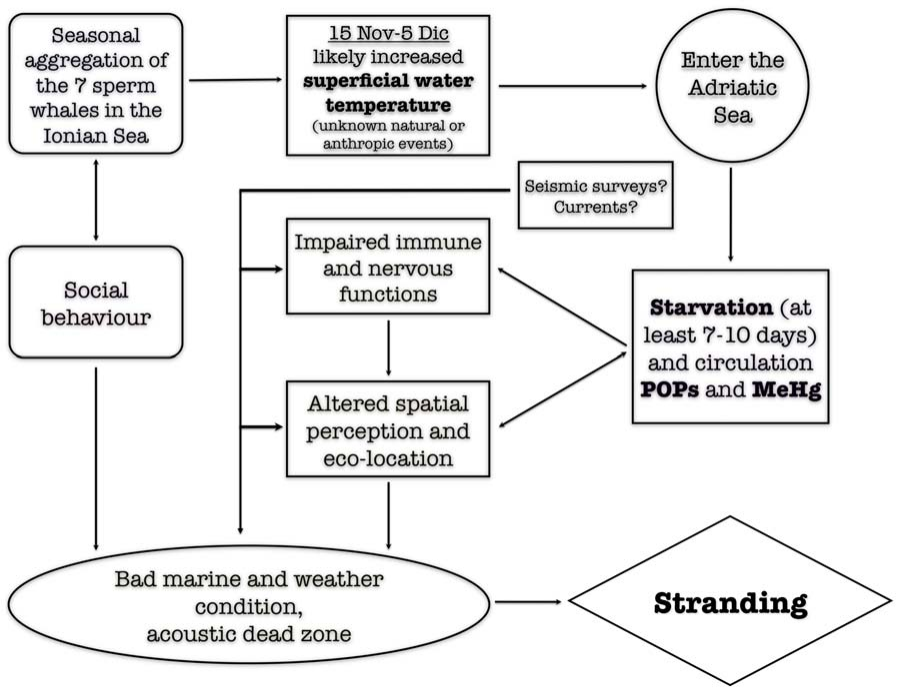
Possible causes and mechanism of stranding. The scheme shows the hypothetical sequence of events that lead the sperm whale pod to strand along the Gargano coastline, with the possible co-factors that could have played a role in determining the event.

In conclusion, we assume these animals took the same wrong way that already lead five other sperm whale pods to strand along the Adriatic Sea coastline in the past. We may conclude that the Eastern South Adriatic coastline is a potentially dangerous trap for sperm whales (Fig. 7).

We emphasize that the results reported herein were obtained through a coordinated and multidisciplinary effort, which unequivocally characterized the postmortem investigations and analyses performed on these whales. This effort suggests that our approach may constitute a practical and systematic example on how to investigate similar mass stranding events.

## Materials and Methods

Our investigations on the seven sperm whales (labeled from no. 1 to no. 7; no. 5, 6 and 7 stranded alive) were performed to evaluate the current hypotheses on the causes of sperm whale mass strandings considering also the impact of human activities in the area. The huge size of the animals, the distance between each individual (whales no. 1 and no. 7 were separated by 3.8 km), the weather conditions and the necessity to establish a safe and healthy working setting, forbade full autopsy of all the seven whales. Before their postmortem examinations, all the 7 animals were measured and the total length was used to estimate weight according to formulas specific for sperm whales [1]. During their disposal, the carcasses and the remains were individually weighted to obtain a “corrected” weight, which considers fluids and tissues lost during *postmortem* and disposal procedures.

As stated above, a detailed postmortem investigation was carried out on three sperm whales. Careful external and gastric inspections were also performed on the bodies of the other four animals. During the three full necropsies, a complete sampling of all organs and tissues was performed by the Necropsy Unit for later microscopic examinations (i.e. histopathology, immunohistochemical and ultrastructural investigations were performed on tissues fixed by immersion in aldehydes), as well as virological, microbiological, and biomolecular investigations for *Toxoplasma gondii* (genomic DNA was extracted from mesenteric, prescapular, and pancreatic lymph nodes, liver and brainstem of whale no. 6, and from prescapular and mesenteric lymph nodes and liver of animal no. 7). Specific searches were started for biotoxins (frozen samples of livers of animals no. 5, 6 and 7 and brainstem of animal no. 6), gas and fat emboli (veins were screened grossly looking for gas emboli and gas bubbles were collected by vacutainer® tubes from the coronary veins; lung tissues fixed in formalin were used to investigate the presence of lipid droplets) and contaminants (performed on frozen livers and kidneys of whales no. 5, 6 and 7).

Additionally, extensive samplings were performed on all the seven animals for age determination (two teeth for each animal), genetic analyses (skin preserved in DMSO and frozen), stable isotope analyses (performed on frozen skin, blubber, liver and muscle), and toxicology (carried out on frozen muscular and adipose tissue samples from the animals of the entire pod). Gastric contents were collected during opening of the stomach complexes: the three components (organic, inorganic, parasites) were separated, weighed and later examined. All the aforementioned biological samples were collected and stored in the Mediterranean Marine Mammals Tissue Bank of the University of Padua, to be subsequently distributed to all other partner Institutions involved in the present study for all further investigations. A more detailed explanation of the laboratory analyses performed on the seven stranded whales is provided below.

### Age determination

One mandibular tooth from each sperm whale was collected during field sampling and subsequently sectioned along the sagittal plane. One half was observed directly, and the other half was polished and then etched in 15% formic acid until clear, easily discernible dentinal layers or growth layer groups (GLGs) were produced. The total number of GLGs in each of the seven tooth sections was determined in three sessions by four independent readers [63].

### Genetics

Skin samples were collected from ten Mediterranean sperm whales (seven stranded in Puglia, two in Sardinia and one in Tuscany), and two from the Sea of Cortez (Mexico). Genomic DNA was extracted from the samples using standard proteinase K digestion and phenol/chloroform extraction protocol [64]. Samples were sexed using ZFX and SRY primers described in previous papers [65]. A 954 bp fragment of the mitochondrial DNA control region was amplified, forward primer was designed on the tRNA Threonine gene, while the reverse primer was designed between the phenylalanine and the 12s rRNA genes. Cycle sequencing products were purified and then sequenced using Big Dye chemistry (Applied Biosystems) and were analyzed in forward and reverse directions on an ABI 3100 sequencer. All sequences were aligned using CodonCode Aligner Software. Genetic analyses were carried out using Arlequin v 3.5.

### Photo-identification

Several photos of the flukes of each whale were taken to record any available characteristic that could result in individual identification, as marks (nicks, notches, scallops, irregularities etc.) along the trailing edge of the flukes and/or pigmentation marks on the ventral or dorsal surface of the flukes. Additional photos of the dorsal fin and lower jaws were taken in case they could provide assistance to the identification process. The best photos of each of the seven stranded whales were compared with all the available photos from three sperm whale photographic databases of free ranging sperm whales of the Mediterranean Sea from the period 1990–2009 (GREPHYSC 2009, Greek Physeter Catalogue; the Mediterranean part of NAMSC 2004, North Atlantic & Mediterranean Sperm Whale Catalogue; the Tethys sperm whale catalogue 2008, TeSC). A non-automatic comparison was performed by visual inspection of each photo, since the total number of known individuals in the Mediterranean Sea is relatively small (few hundreds).

### Stable isotope analysis

Stable isotopes (δ^13^C and δ^15^N) were analyzed in several tissues (blubber, skin, liver and muscle). Lipids were removed from all samples using a mixture of chloroform and methanol (1∶1). Samples were then dried at 60°C and grounded into a homogeneous fine powder with mortar and pestle. Isotopic analyses were completed by the Stable Isotope Laboratory at the University of Palermo using a Thermo Scientific isotope ratio mass spectrometer (DeltaPlus XP) interfaced in continuous flow to a Thermo Scientific Elemental Analyzer (EA 1112). Isotopic values were expressed in δ notation as parts per thousand (‰) differences from international standards (Vienna Pee Dee Belemnite and atmospheric N_2_ for carbon and nitrogen respectively): δX = [(R_sample_/R_standard_)−1]×10^3^, where X is equal to ^15^N or ^13^C and R is the ratio ^13^C/^12^C or ^15^N/^14^N. Based on replicates of laboratory standards, analytical precision was ±0.2 and ±0.1‰ for δ^15^N and δ^13^C, respectively.

### Microscopic analyses (histopathology, immunohistochemistry, and ultrastructural investigations)

Tissues samples collected from all organs, as well as from grossly evident lesions, of the three whales who underwent a full necropsy were formalin-fixed, paraffin-embedded, cut into 5 µm thick sections and then stained with hematoxylin and eosin for routine light microscopic evaluation. Periodic acid Schiff (PAS), Gram and Giemsa stainings were also performed to assess the evidence of any biological agent within the tissues. Selected tissue sections from lungs and lymph nodes from animals no. 5, 6 and 7, along with the brainstem from whale no. 6, were submitted to a detailed search for *Morbillivirus* antigen by means of a proper immunohistochemical technique reported elsewhere [66].

Auto-methallographic techniques, known also as Danscher's staining [67], were employed to assess the presence of inorganic mercury deposition within the cells, along with X-ray microanalysis that was performed using an Environmental Scanning Electron Microscopy (ESEM), coupled with a fluorescence X-ray scanning system. Schmorl's stain was used to evaluate the presence of lipofuscin pigments. Finally, post-fixation of lung samples stored in buffered formalin in osmium tetroxide (OsO_4_) was employed to detect fat emboli [68].

### Gastric content analyses

Cephalopod beaks exclusively represented the organic fraction of the stomachs contents. They were washed, weighed and sorted according to three stages of digestion: complete, damaged, and highly digested beaks. Only the first two stages were taken into account and divided into upper and lower beaks for counting. The lower beaks were identified to the lowest taxonomic level possible, according to Clarke (1986) [69]. The foreign bodies were also weighed and sorted.

### Parasitology

A detailed parasitological survey was also carried out on three out of the seven stranded sperm whales. Macro-parasites were collected, stored in 70% ethanol, and morphologically studied by light microscope and stereomicroscope.

DNA extracts were subjected to a nested PCR specific for the B1 gene of *Toxoplasma gondii* [70]. This gene was selected being a repetitive DNA sequence of *T. gondii* mostly used for the detection of the protozoan by nested PCR on biological samples for diagnostic purposes [71]. A region of ∼100bp within the B1 gene was amplified with the primer pair B1outF (5′-GGAACTGCATCCGTTCATGAG-3′) and B1outR (5′-TCTTTAAAGCGTTCGTGGTC-3′) in a first round of PCR, followed by a second round using the primer set B1intF (5′-TGCATAGGTTGCAGTCACTG-3′) and B1intR (5′-GGCGACCAATCTGCGAATACACC-3′). Both amplification rounds for B1 gene consisted of an initial step of 10 min at 95°C, 35 cycles, each of 60 sec at 94°C, 60 sec at 50°C (first step) or 54°C (second step), 60 sec at 72°C, with a final extension of 10 min at 72°C. In the first step the mixture (50 µl) consisted of 4 µl of DNA extract, 50 pmol of each of the primers and 25 µl of Ready Mix REDTaq (Sigma, St. Louis, MO). The conditions for the second step mixture were identical to those for the primary PCR, with the exception of the template amount (i.e. 4 µl of a 1/10 dilution of the primary B1 PCR product, determined to be optimal). Successful PCR products were purified directly and sequenced using a Taq DyeDeoxyTerminator cycle sequencing kit in an ABI-PRISM model 377 sequencer (Perkin Elmer, Warrington, UK). Accuracy was achieved by two-directional sequencing and all electropherograms were manually checked and edited. Sequences obtained were compared with sequences registered in the GenBank™ for each gene examined using the Nucleotide-Nucleotide “Basic Local Alignment Search Tool” [72].

### Biotoxins and ecotoxicology

#### a. Biotoxins

Lipophilic (okadaic acid, dinophysistoxins, pectenotoxins, yessotoxins, adriatoxin and azaspiracids) and hydrophilic (paralytic shellfish poisoning, domoic acid and brevetoxins) biotoxins were assessed by High Performance Liquid Chromatography (HPLC) analysis in livers of whales no. 5, 6 and 7 and in the brainstem of no. 6, according to the procedures detailed the EU legislation regulating the detection of marine toxins in seafood (Decision 2002/225/EC).

#### b. Analytical chemistry-heavy metals

Evaluation of heavy metal concentrations, and in particular total mercury (TotHg), Se and Cd, was performed using an atomic absorbance spectrophotometer in muscles of all the animals, and in the kidney and liver of animals no. 5, 6 and 7 by using about 200mg of lyophilized material digested in a Teflon bomb with 0,2 ml of nitric acid. The solution obtained was analyzed by atomic absorption spectroscopy. The cold vapor technique was used for mercury and the volatile hydrides method for selenium. The graphite furnace atomizer was used for cadmium. Organic mercury (in particular methyl-mercury, MeHg) was studied on livers, kidneys and muscles of whales no. 5, 6 and 7, prescapular lymph nodes of animals no. 5 and 6, and brainstem of no. 6 by isolating it from tissues with the addition of HCl, followed by extraction with toluene. Quantification was carried out by gas chromatography/mass spectrometry with an average recovery value of 97%.

#### c. Analytical chemistry-lipid extraction

Blubber samples were freeze-dried and extracted with n-hexane in a Soxhlet apparatus. The lipid content of the extracted organic material was determined by gravimetry.

#### d. Analytical chemistry-PAHs

PAHs were analyzed by HPLC with fluorescence detection [73]. The samples of blubber, liver and muscle (about 2g) were lyophilized in an Edwards freeze drier for 2 days and extracted according to Griest and Caton (1983) [74] with modifications [75] and samples were concentrated to 1 ml in acetonitrile. A reverse-phase column (Supelcosil LC-18, 25 cm×4.6mm i.d., 0.5 µm particle size) and an acetonitrile/water gradient were used. Initial gradient was 60% acetonitrile and increased to 100% over 20min, then remaining stable for 10 min. Flow-rate was 1 ml/min. The external standard consisted of 16PAHs from Supelco (EPA 610 PAH mixture). Results were expressed as the sum of 14 PAHs (naphtalene, acenaphtene, fluorene, phenanthrene, anthracene, fluoranthene, pyrene, Benzo(a)anthracene, chrysene, benzo(b)fluoranthene, benzo(k)fluoranthene, benzo(a)pyrene, dibenzo(ah)anthracene, benzo(ghi)perylene) per ng/g on a lipid basis. Assay reproducibility was determined by five repeated analyses (variation coefficient ranged 1–3%), recoveries of standard ranged 80–98%, and no PAHs were detected in blanks.

#### e. Analytical Chemistry-OCs

Analyses for HCB, DDTs and PCBs were performed according to methods recommended by the U.S. Environmental Protection Agency (EPA) 8081/8082 with modifications [75]. The samples of blubber, liver and muscle (about 2 g) were lyophilized in an Edwards freeze drier for 2 days and extracted with n-hexane for gas chromatography (Merck) in a Soxhlet apparatus for analysis of organochlorinated compounds. Whatman® cellulose thimbles (i.d. 25 mm, e.d. 27 mm, length 100 mm) to be used for extraction of the samples were preheated for about 30 min to 110°C and pre-extracted for 9 h in a Soxhlet apparatus with n-hexane, to remove organochlorinated contamination. Each sample was spiked with surrogate compound (2,4,6-trichlorobiphenyls - IUPAC number 30) [76] prior to extraction. This compound was quantified and its recovery calculated. Surrogate recovery was reported with the sample results. The samples were then purified with sulphuric acid to obtain a first lipid sedimentation. The extract then underwent liquid chromatography on a column containing Florisil that had been dried for 1 h in an oven at 110°C. This further purified the apolar phase of lipids that could not be saponified, including steroids like cholesterol. Decachlorobiphenyl (DCBP - IUPAC number 209) was used as an internal standard, added to each sample extract prior to analysis, and included in the calibration standard, a mixture of specific compounds (Aroclor 1260, HCB and pp′- and op′-DDT, DDD and DDE). The analytical method used was High Resolution Capillary Gas Chromatography with an Agilent 6890N and a 63Ni ECD and an SBP-5 bonded phase capillary column (30 m long, 0.2 mm i.d.). The carrier gas was N_2_ with a head pressure of 15.5 psi (splitting ratio 50/1). The scavenger gas was argon/methane (95/5) at 40 ml/min. Oven temperature was 100°C for the first 10 min, after which it was increased to 280°C at 5°C/min. Injector and detector temperatures were 200°C and 280°C respectively. The extracted organic material (EOM%) from freeze-dried samples was calculated in all samples. Capillary gaschromatography revealed op′- and pp′- isomers of DDT and its derivatives DDD and DDE, and about 30 PCB congeners. Total PCBs were quantified as the sum of all congeners. These congeners constituted 80% of the total peak area of PCBs in the samples. Total DDT was calculated as the sum of *op′*DDT, *pp′*DDT, *op′*DDD, *pp′*DDD, *op′*DDE and *pp′*DDE. The results were expressed in ng/g lipidic weight (ng/g lwt)

#### f. Statistical analysis

To compare the levels in the tested biological matrixes of the three considered xenobiotics, the non-parametric test of Kruskal-Wallis was used, using also the Kolmogorov-Smirnov test to evaluate any significant difference (p<0.025).

#### g. Biological trials

Biological effects due to any of the chemical substances found during toxicological investigations on sperm whales were investigated using 80 mosquito fishes (*Gambusia affinis*) divided into four experimental groups of 20 animals each: two were exposed to liquid liver extracts obtained from animals no. 6 and no. 7, while another group was exposed to extracts of porcine liver. A fourth group of 20 animals living in non-contaminated water was used as control. All the groups were maintained in similar environmental conditions and continuously monitored. Toxicity was investigated after 48 hours of exposure by evaluating the activity of several biomarkers: erythrocytic nuclear abnormalities (ENA) and Comet Assay, to study any genotoxic effect; acetyl-cholinesterase (AChE) to investigate neurotoxicity; ethoxyresorufin-O-deethylase (EROD) to assess the presence of lipophilic toxins.

### Meteorological and stranding data sets

In the present study, a multidisciplinary and wide range investigation was performed to assess any of the possible causes reported as temporally and spatially associated to mass strandings of sperm whales. Weather and marine data were obtained from different national and international meteorological archives, namely the Institute for the Protection of the Environment and Research (ISPRA, www.idromare.it), the Marine Forecasting Systems National Group of Operational Oceanography of the National Institute for Geophysics and Vulcanology (GNOO-INGV, www.gnoo.bo.ingv.it), the European Ocean Observatory Network (data from the observatory E2-M3A of the project EuroSITES, www.eurosites.info), and the “Poseidon System” of the Hellenic Center for Marine Research (www.poseidon.hcmr.gr).

Previous surveys reported solar activity and lunar cycles among those natural factors potentially causing and/or influencing sperm whale mass stranding events, thus prompting the search for relevant anomalies in our scenario. The INGV also provided us with information on past seaquakes and geomagnetic field abnormalities (www.earthquake.rm.ingv.it), while information on solar activity and cycles was obtained from the US National Oceanic and Atmospheric Administration (NOAA, www.swpc.noaa.gov). Any military activity or authorized seismic surveys in the surrounding waters were looked for in the governmental and official websites.

Finally, historical data sets on cetacean strandings along the Southern Adriatic Sea coastline, as well as those specifically concerning sperm whales in Italy, were obtained from the Italian Stranding Database (www.mammiferimarini.unipv.it/).
